# Revisional Roux-en-Y Gastric Bypass Versus Revisional One-Anastomosis Gastric Bypass After Failed Sleeve Gastrectomy: a Randomized Controlled Trial

**DOI:** 10.1007/s11695-022-06266-8

**Published:** 2022-09-13

**Authors:** Mohamed Hany, Ahmed Zidan, Ehab Elmongui, Bart Torensma

**Affiliations:** 1 Department of Surgery, Medical Research Institute, Alexandria University, 165 Horreya Avenue, Hadara, Alexandria, 21561 Egypt; 2 Consultant of Bariatric Surgery, Madina Women’s Hospital (IFSO Center of Excellence), Alexandria, Egypt; 3 Health Insurance Organization, Alexandria, Egypt; 4 Leiden University Medical Center (LUMC), Leiden, The Netherlands

**Keywords:** Laparoscopic sleeve gastrectomy, Weight regain, Revisional bariatric surgery, Roux-en-Y gastric bypass (RYGB), One-anastomosis gastric bypass (OAGB)

## Abstract

**Background:**

High rates of revision surgery have been reported for laparoscopic sleeve gastrectomy (LSG), with weight regain (WR) as the most frequently reported cause. Roux-en-Y gastric bypass (RYGB) is the most commonly performed revision procedure, whereas one-anastomosis gastric bypass (OAGB) is a less popular approach.

**Methods:**

A single-blinded randomized controlled trial was conducted. One hundred seventy-six patients were enrolled and randomized. After loss to follow-up, 80 patients for RYGB and 80 patients for OAGB were analyzed, with a 2-year follow-up. Patients with grade B or higher gastroesophageal reflux disease (GERD) were excluded. Early and late postoperative complications were recorded. Body mass index (BMI), percentage of excess BMI loss (%EBMIL), nutritional laboratory test results, and the resolution of associated medical problems were assessed after revision surgery.

**Results:**

After 2 years, both groups achieved significantly lower BMI than their post-LSG nadir BMI (*p* < 0.001). The %EBMIL changes showed significantly faster weight loss in the OAGB group than in the RYGB at the 6-month follow-up (mean difference: 8.5%, 95% confidence interval [CI]: 0.2 to 16.9%). However, at 1-year and 2-year follow-ups, the differences were statistically insignificant (*p* > 0.05). Early and late complications were similar between two groups. Both groups showed improvement or resolution of associated medical problems, with no statistically significant differences after 2 years (*p* = 1.00).

**Conclusion:**

Both revisional RYGB and OAGB have comparable significant weight loss effects when performed for WR after LSG. After a 2-year follow-up, both procedures were safe, with no significant differences in the occurrence of complications and nutritional deficits.

**Graphical abstract:**

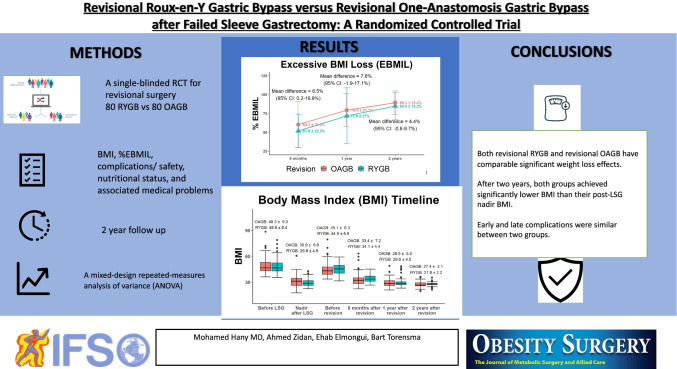

## Introduction

After being introduced as a first-step procedure in the biliopancreatic diversion with duodenal switch (BPD-DS), laparoscopic sleeve gastrectomy (LSG) has been proven as a safe stand-alone bariatric procedure with low complication rates and excellent short-term weight loss [1–3]. Since 2014, LSG has gained popularity and become the most frequently performed bariatric procedure, accounting for up to 55% of all bariatric procedures performed worldwide in 2018 [4]. Highly variable rates of conversion of LSG to other bariatric procedures have been reported in the literature, ranging from 2.5 to 33% [3, 5]. In this regard, weight regain (WR) and weight loss failure (WLF) accounted for 70% of post-LSG revision surgeries [3, 5, 6]. Gastroesophageal reflux disease (GERD) is the second most commonly reported cause of revision [3, 5]. The results showed that revision LSG surgery showed better weight loss than primary LSG [3]. Roux-en-Y gastric bypass (RYGB) is the most commonly performed revision procedure after LSG, followed by re-sleeve, while one-anastomosis gastric bypass (OAGB) is a less common approach [3, 5]. Primary OAGB has gained popularity over the last decade and is currently the third most common bariatric procedure after LSG and RYGB. The safety and weight loss results of OAGB were reportedly equal or higher than those of LSG and RYGB [4, 7]. The revision of OAGB after LSG also showed excellent results regarding weight loss and the resolution of associated medical problems [7, 8].

Data on OAGB as a revision surgery for failed LSG are scarce. To our best knowledge, no published randomized controlled trials have compared revisional RYGB and OAGB as post-LSG options. This study aimed to compare the outcomes of RYGB and OAGB after failed LSG in terms of weight loss, occurrence of complications, resolution of associated medical problems, and nutritional assessment over 2 years of follow-up.

## Methods

### Study Design

This single-blinded randomized controlled trial aimed to compare the outcomes between revisional RYGB and OAGB for weight regain after LSG during 2 years of follow-up between August 2018 and February 2020 at the Medical Research Institute, Alexandria University, Egypt. The study protocol was approved and registered with the Ethics Committee of Alexandria University by an institutional review board with the registration number: *(R.172335)*. The CONSORT checklist was used (Appendix Table 4) [9]. Before trial participation, differences in the expected benefits and possible risks of the two procedures were explained to the patients. All participants signed an informed consent form before enrolling in this study. The revision procedures were performed at two specialized bariatric centers. Two main/primary surgeons performed all the procedures with four assistant surgeons. Both main/primary surgeons performed both RYGB and OAGB procedures. They were the surgeons for original LSG in around half of the cases, while the other cases were referred from other centers.

### Inclusion and Exclusion Criteria

Inclusion criteria were as follows: age between 18 and 60 years. Weight regain was the main inclusion criterion, defined as any increase in weight above the nadir as reported by the patient [10, 11]. The mean BMI at the time of revisional surgery was around 45 kg/m^2^, which is an indication of bariatric surgery. Moreover, regarding BMI, weight regain was defined as an increase in BMI after bariatric surgery to exceed 35 [11]. Furthermore, all patients had routine per-operative gastroscopy (EGD). Patients with grade B or higher GERD according to the Los Angeles (LA) classification [12] were excluded from the study.

### Study Endpoints

Study endpoints include weight loss, the occurrence of complications, nutritional laboratory test results, and resolution and/or improvement of associated medical problems after the weight loss. [13]

### Data Collection

Preoperative data, such as patient’s demographics, body mass index (BMI), time between the primary and revision procedures, associated medical problems, endoscopy and imaging findings, sleeve volume assessed by multi-detector computed tomography (MDCT) virtual gastroscopy, and laboratory investigations, were collected. Furthermore, an assessment by a multi-disciplinary team of dietician, psychiatrist, and medical bariatric surgical nursing in the out-patient clinic was done.

Perioperative data included operation time and combined operative procedures, such as hiatal hernia repair and cholecystectomy. Postoperative data included early and late complications, nutritional laboratory results, reflux symptoms (including heartburn, acid or bile reflux to the mouth, and retrosternal pain), changes in BMI and percentage of excess BMI loss (%EBMIL) at 6-month, 1-year, and 2-year follow-ups, and resolution or improvement of associated medical problems at 2 years after revision surgery.

### Preoperative Care

A multidisciplinary team (MDT) evaluation was arranged for all patients. The MDT included a bariatric surgeon, a dietician, an internist, and a psychiatrist. The dietician assessed the defects in the patient’s nutrition, explained the expected nutrition problems after surgery, and started instructing the patients about the proper regimens to achieve good weight loss after surgery. The internist assessed the patients for chronic associated medical problems such as diabetes mellitus and hypertension. The psychiatrist identified the patients with eating disorders like emotional eating or patients who needed psychological support, perioperatively.

Laboratory assessment included routine and nutritional tests for all patients. Abdominal ultrasound (U/S) examination was routinely performed. Upper gastrointestinal endoscopy was routinely done; the presence of hiatal hernia was determined by endoscopy; and GERD was evaluated using the LA classification [12].‏

### Surgical Techniques

The RYGB and OAGB procedures were performed by the same surgeons and laparoscopies with standard five ports. A full description of the techniques is provided in Appendix 2.

### Postoperative Care

Prophylaxis against venous thrombosis was started 12 h before surgery with enoxaparin and was continued for 21 days after surgery. A routine gastrografin swallow was performed on day 1 after surgery. MDCT with intravenous (IV) and oral contrast were performed when patients had alarming complaints of complications, such as tachycardia, persistent abdominal pain, fever, persistent vomiting, and abdominal distension. Endoscopy was not routinely done after surgery; it was performed in patients with persistent epigastric pain, heartburn, regurgitation, bilious vomiting, interruption of sleep, dyspepsia, or melena. Diagnosis of GERD was determined according to the LA classification [12], while bile reflux was diagnosed by visualization of bile in the gastric pouch and esophagus during endoscopy. Multi-vitamins, calcium, and iron supplements were prescribed to all patients.

### Follow-up During the COVID-19 Pandemic

A combination of online virtual (when possible) and physical (with special time slots so that only one patient was present at a time) follow-ups were conducted.

### Statistical Analysis

For the analyses, we used descriptive and inferential statistics. All data were first tested for normality using the Kolmogorov–Smirnov test, Q-Q plot, and Levene’s test. Categorical variables were expressed as *n* (%). Continuous normally distributed variables were expressed with their means and standard deviations, while non-normally distributed variables were expressed with their medians and interquartile ranges. Categorical variables were tested using Pearson’s chi-squared test or Fisher’s exact test, when appropriate. Continuous normally distributed data were tested with the Student’s *t*-test for independent samples. For non-normally distributed data, the Mann–Whitney *U*-test was used for independent samples.

A mixed-design repeated-measures analysis of variance (ANOVA) was used to compare the %EBMIL and BMI within each group at different time points. Adjusted post hoc pairwise comparisons were conducted using the Bonferroni test. Statistical significance was set at *p* ≤ 0.05. Statistical analyses were performed using the R software (version 4.1.3. package).

### Sample Size

Sample size calculation was performed using the R software (version 4.1.3 and its “pwr” package). A medium effect size of 0.5, corresponding to a mean difference in %EBMIL between OAGB and RYGB of at least 10%, was used as the main endpoint. A power of 80% with an alpha of 0.05 was used, resulting in a minimum sample size of 64 patients per group. Considering a possible loss of patients to follow-up, an additional 20% increase in sample size was included, resulting in a total minimum of 154 patients.

### Randomization

A single-blind randomization procedure, in which patients and outpatient clinic nurses were blinded to the study period, was performed. The surgeon was not blinded and received the allocation after the patient was under anesthesia. Randomization was done with randomized block randomization using computer-generated blocks of 4 or 8 block sizes.

### Data Capture

The analysis was performed on a blinded dataset after the completion of medical/scientific review. All protocol violations were identified and resolved, and the dataset was declared complete. All data were collected in a data management system (Castor EDC, Amsterdam, The Netherlands; https://www.castoredc.com) and handled according to Good Clinical Practice guidelines, Data Protection Directive certificate, and complied with Title 21 CFR Part 11. Furthermore, the data centers, where all the research data were stored, were certified according to ISO27001, ISO9001, and Dutch NEN7510.

## Results

### Baseline Characteristics

**Fig. 1:**
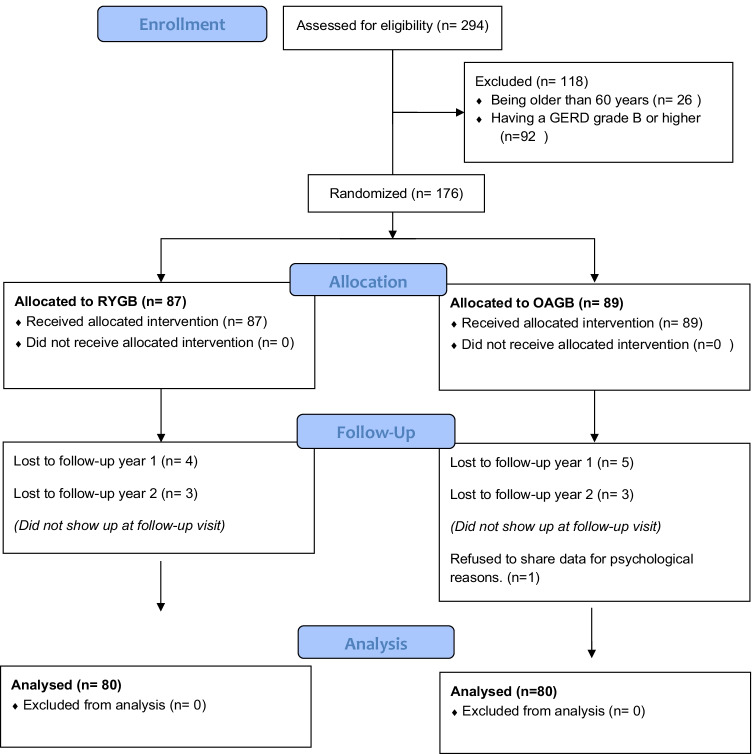
CONSORT 2010 flow diagram

This single-blinded, randomized controlled trial included at the start 176 patients, 87 in the RYGB, and 89 in the OAGB group. During the patient’s enrollment in the study, 294 patients with previous LSG had revisional RYGB or OAGB; 26 were excluded from the study for being older than 60 years; and 92 patients were excluded for having a GERD of grade B or higher. After 1 year, due to loss of follow-up, from the 176 patients, 83 were left in the RYGB, and 84 were in the OAGB group. In year two, 80 patients were left in RYGB and 81 in the OAGB group. One patient was excluded from the OAGB group since this patient refused to share data for psychological reasons. The analysis was done over 160 patients, with 138 (86.3%) women. There were 80 patients in the RYGB group and 80 in the OAGB group. (CONSORT flow diagram, Fig. 1) [9]. No significant differences were present in baseline characteristics, including demographic data, associated medical problems, pre-revision imaging, and laboratory results. The mean age was 43.0 ± 7.2, and the main BMI was 45.0 ± 7.5 (Table 1).

**Table 1:** Baseline characteristics

	OAGB *N* = 80	RYGB *N* = 80	*p*-Value
Age (years)	42.6 ± 7.1	43.4 ± 7.5	0.490
Sex (female) *n* (%)	69 (86.3%)	69 (86.3%)	1
BMI before 1 ry LSG kg/m^2^	49.3 ± 9.0	48.8 ± 8.4	0.713
Nadir BMI after 1 ry LSG kg/m^2^	30.9 ± 6.6	29.8 ± 4.9	0.231
BMI before revision kg/m^2^	45.1 ± 8.3	44.9 ± 6.6	0.904
Mean time between LSG and revision (years)	5.9 ± 1.3	6.0 ± 1.3	0.584
Smoking *n* (%)	7 (8.8%)	7 (8.8%)	1
Associated medical problems *n* (%)			
Hypertension	11 (13.8%)	14 (17.5%)	0.66
Diabetes mellitus	12 (15.0%)	6 (7.5%)	0.21
Obstructive sleep apnea	6 (7.5%)	7 (8.8%)	1
Dyslipidemia	18 (22.5%)	16 (20.0%)	0.85
Osteoarthritis	3 (3.8%)	4 (5.0%)	1
Cardiac disease	2 (2.5%)	1 (1.3%)	1
Menstrual irregularities	5 (6.3%)	3 (3.8%)	0.72
Asthma	2 (2.5%)	3 (3.8%)	1
Imaging before revision			
Mean sleeve pouch volume (mL)	648.5 ± 101.7	620.9 ± 93.6	0.076
Hiatal hernia *n* (%)	46 (57.5%)	56 (70.0%)	0.14
Chronic calcular cholecystitis	11 (13.8%)	19 (23.8%)	0.16
Endoscopy before revision *n* (%)			
Hiatal hernia	46 (57.5%)	56 (70.0%)	0.14
GERD grade A	33 (41.3%)	32 (40.0%)	1
Pre-revision lab investigations			
Hemoglobin (gm/dL)	13.6 ± 1.9	13.5 ± 2.2	0.819
WBCs (10^9^/L)	7.3 ± 2.2	7.6 ± 1.7	0.391
Ferritin (mcg/dL)	128.2 ± 71.0	127.9 ± 67.8	0.982
SGOT (U/L)	20.9 ± 7.6	23.5 ± 7.3	**0.032**
SGPT (U/L)	29.5 ± 12.0	28.8 ± 11.4	0.718
Urea (mg/dL)	27.1 ± 7.1	28.9 ± 8.6	0.164
Creatinine (mg/dL)	0.8 ± 0.2	0.8 ± 0.2	0.840
INR (units)	1.0 ± 0.1	1.0 ± 0.1	0.774
T3 (mcg/dL)	3.0 ± 0.8	2.9 ± 1.0	0.376
T4 (mcg/dL)	1.3 ± 0.5	1.1 ± 0.5	**0.006**
TSH (mlU/mL)	2.7 ± 1.3	2.4 ± 1.4	0.198
Fasting blood glucose (mg/dL)	96.3 ± 15.8	94.8 ± 14.2	0.512
HbA1c (mg/dL)	5.0 ± 1.5	4.9 ± 1.2	0.512
Cholesterol (mg/dL)	200.0 ± 67.4	200.0 ± 68.7	0.995
Triglycerides (mg/dL)	141.6 ± 30.1	140.2 ± 31.1	0.769
LDL (mg/dL)	92.4 ± 41.3	85.4 ± 30.4	0.228
Albumin (mg/dL)	4.0 ± 0.5	4.0 ± 0.5	0.527
Calcium (mg/dL)	8.9 ± 1.1	8.9 ± 1.1	0.956
Vitamin D3 (ng/mL)	34.1 ± 11.4	33.8 ± 12.3	0.849
Vitamin B12 (pg/mL)	446.1 ± 240.0	451.4 ± 245.0	0.890
Parathormone (pg/mL)	37.6 ± 12.6	38.5 ± 11.7	0.646

Significance p ≤ 0.05

### Virtual Follow-up Visits

Due to the COVID crisis, the government implemented a nationwide lockdown in March 2020 and started to lift the restrictions on 27 June 2020 partially; during the lockdown time, 92% of the appointments were virtual and kept a high rate above 80% until April 2021; after that, the rate of virtual appointments had a gradual reduction till it reached 10% in early 2022.

### Operative and Postoperative Data

Operation time was significantly lower in the OAGB group than in the RYGB (85.6 ± 18.6 versus 104.9 ± 13.7 min, *p* < 0.001). In total, one (1.3%) incidence of leakage at the esophagogastric junction (EGJ) was detected in the RYGB group (*p* = 0.32), which was managed conservatively. In each group, two cases of bleeding were observed (*p* = 1.00). Partial portal vein thrombosis occurred once in the OAGB group (*p* = 0.32) (Table 2). No mortality was detected in both cohorts of the study.

**Table 2:** Operative and post-operative data

	OAGB *N* = 80	RYGB *N* = 80	*p* Value
Operative time (min)	85.6 ± 18.6	104.9 ± 13.7	** < .001**
Combined surgery *n* (%)			
Cholecystectomy	3 (3.8%)	7 (8.8%)	.19
Hiatal hernia repair	27 (33.8%)	28 (35.0%)	.87
Cholecystectomy and hiatal hernia repair	9 (11.3%)	12 (15.0%)	.48
Early complications *n* (%)			
Leak	0 (0.0%)	1 (1.3%)	.32
Bleeding	1 (1.3%)	1 (1.3%)	1
Portal vein partial thrombosis	1 (1.3%)	0 (0.0%)	.32
Melaena	0 (0.0%)	1 (1.3%)	.32
Wound infection	1 (1.3%)	0 (0.0%)	.32
Late complications *n* (%)			
Internal hernia	0 (0.0%)	1 (1.3%)	.32
Port-site hernia	1 (1.3%)	1 (1.3%)	1
Marginal ulcers	0 (0.0%)	2 (2.5%)	.16
Post-operative endoscopy *n* (%)			
Hiatal hernia	3 (3.8%)	2 (2.5%)	.65
Reflux (bile/acid)	2 (2.5%)*	1 (1.3%)**	.56
Marginal ulcers	0 (0.0%)	2 (2.5%)	.16

^*^Bile reflux, **acid reflux (GERD grade A)

Significance ≤ 0.05

### Late Complications

In the RYGB group, internal hernia occurred once (*p* = 0.32), and marginal ulcers occurred twice (*p* = 0.16). Post-site hernia was detected in both groups once (*p* = 1.00).

### Postoperative Endoscopy

De novo grade A GERD was diagnosed in one (1.3%) patient with RYGB (acid reflux), while the bile reflux was diagnosed in two (2.5%) patients in the OAGB group (*p* = 0.56).

### Postoperative Laboratory and Nutritional Assessment

No statistically significant differences were observed between the two groups after 2 years. Hemoglobin A1c (HbA1c, 4.5% ± 0.7 versus 4.8% ± 0.7, *p* = 0.025), and calcium (8.2 mg/dl ± 1.1 versus 8.6 mg/dl ± 1.1, *p* = 0.025) levels were significantly different between two groups. However, these differences were not clinically relevant (Table 3).

**Table 3:** Second-year post-operative follow up lab investigations

	OAGB *N* = 80	RYGB *N* = 80	*p* Value
Hemoglobin (gm/dL)	11.8 ± 1.9	12.0 ± 2.2	0.483
Ferritin (mcg/dL)	115.7 ± 73.5	122.0 ± 84.2	0.616
Fasting blood glucose (mg/dL)	88.0 ± 11.1	89.8 ± 12.4	0.346
HbA1c (mg/dL)	4.5 ± 0.7	4.8 ± 0.7	**0.025**
Cholesterol (mg/dL)	162.2 ± 24.9	160.6 ± 23.1	0.665
Triglycerides (mg/dL)	127.0 ± 19.6	127.3 ± 20.1	0.927
LDL (mg/dL)	71.3 ± 6.8	69.7 ± 6.4	0.137
Albumin (gm/dL)	3.6 ± 0.7	3.7 ± 0.8	0.190
Calcium (mg/dL)	8.2 ± 1.1	8.6 ± 1.1	**0.025**
Vitamin D3 (ng/mL)	30.6 ± 13.0	33.1 ± 12.7	0.229
Vitamin B12 (pg/mL)	405.4 ± 242.5	419.7 ± 237.3	0.706
Parathormone (pg/mL)	37.7 ± 12.6	38.1 ± 11.7	0.841

Significance p ≤ 0.05

### BMI Timeline and %EBMIL

**Fig. 2:**
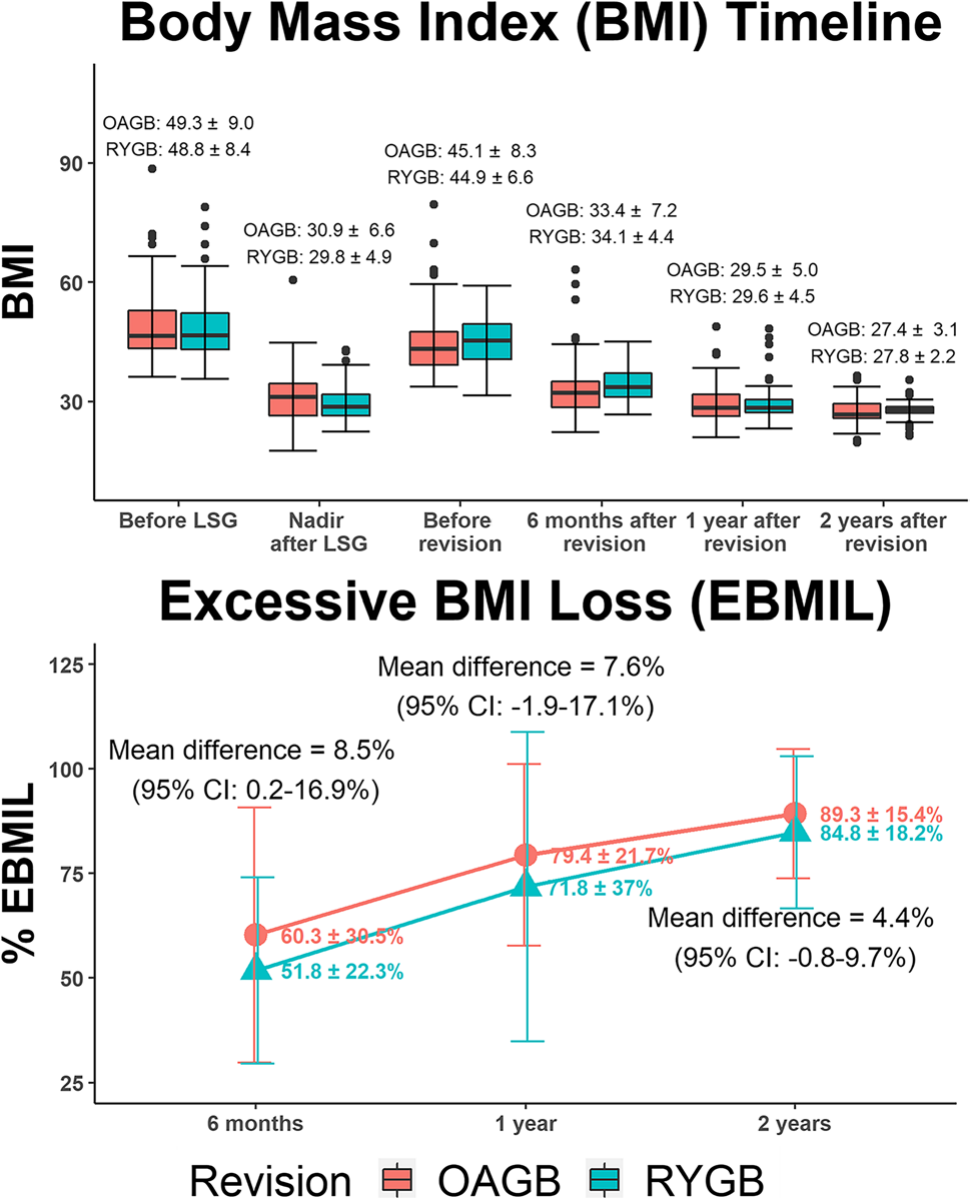
BMI timeline and EBMIL after revisional surgery

BMI values before LSG, after 1 year of LSG, and before revision were 49.1 ± 8.7 kg/m^2^, 30.4 ± 5.8 kg/m^2^, and 45.0 ± 7.5 kg/m^2^, respectively, with a mean time between LSG and revision of 5.9 ± 1.3 years. Nadir BMI after LSG was 30.9 ± 6.6 kg/m^2^ in the OAGB group and 29.8 ± 4.9 kg/m^2^ in the RYGB group. In both groups, BMI significantly changed after revision surgery (*p* < 0.001). At 6 months after revision surgery, BMI values in both groups were significantly higher than their nadir values at 1 year after LSG (OAGB, mean difference: + 2.5 kg/m^2^, *p* = 0.04; RYGB, mean difference: + 4.3 kg/m^2^, *p* < 0.001). At 1 year after revision surgery, BMI values were lower than their post-LSG nadir values in both groups, with no statistically significant difference (OAGB, mean difference: − 1.4 kg/m^2^, *p* = 0.90; RYGB, mean difference: − 0.2 kg/m^2^, *p* = 1.00). At 2 years after revision surgery, both groups showed significantly lower BMI values than their post-LSG nadir values (OAGB, mean difference: − 3.5 kg/m^2^, *p* < 0.001; RYGB, mean difference: − 2.0 kg/m^2^, *p* < 0.001) (Fig. 2).

### %EBMIL

There were no statistically significant differences in the absolute BMI values between the OAGB and RYGB groups at 6 months, 1 year, and 2 years after revision surgery. At 6 months after revision surgery, the %EBMIL in the OAGB group was significantly higher than that in the RYGB group (mean difference: 8.5%, 95% confidence interval [*CI*]: 0.2 to 16.9%). However, the differences between two groups continued to decrease and were no longer statistically significant at 1-year (mean difference: 7.6%, 95% *CI*: − 1.9 to 17.1%) and 2-year (mean difference: 4.4%, 95% *CI*: − 0.8 to 9.7%) follow-ups (Fig. 2).

### Associated Medical Problems

**Fig. 3:**
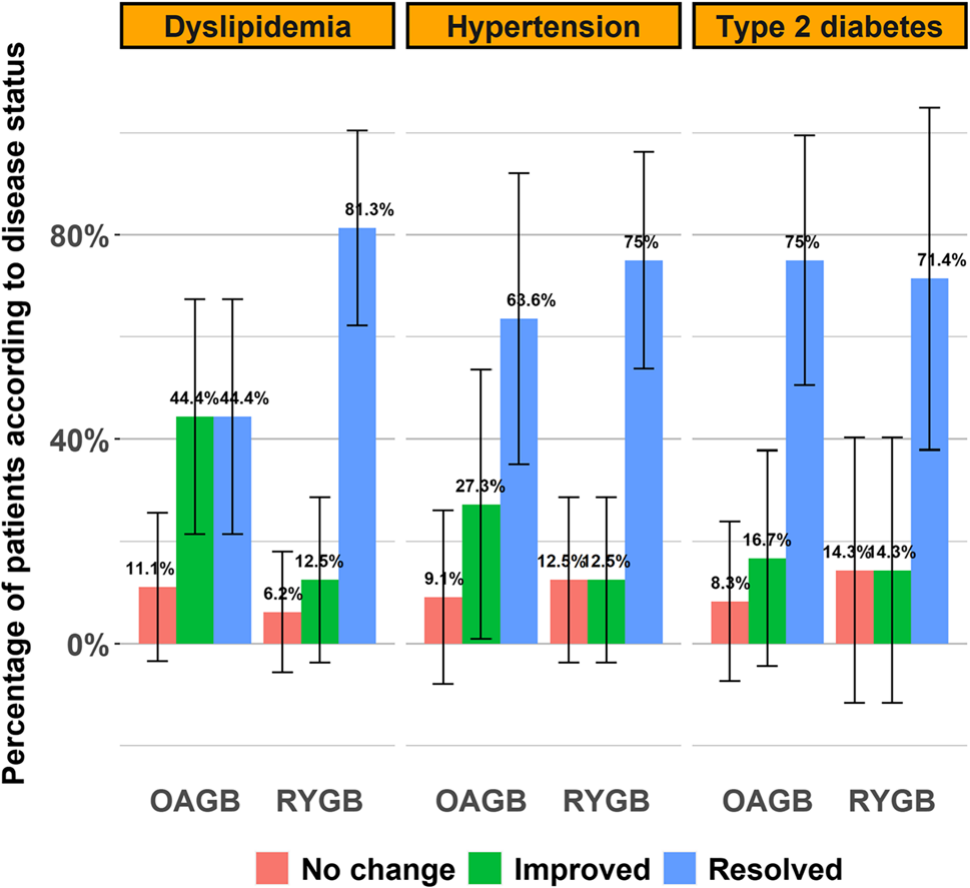
Associated medical conditions evaluation after 2 years

Compared to the preoperative period, both groups showed significant improvement or resolution of dyslipidemia, hypertension, and type 2 diabetes 2 years after revision surgery (*p* < 0.05). However, no significant differences between the two groups were observed (*p* = 1.00) (Fig. 3).

## Discussion

This single-blinded randomized controlled trial compared the outcomes of revisional RYGB and revisional OAGB for weight regain after LSG throughout 2 years of follow-up. WR is the main reason for revision after LSG [3, 14, 15]. WLF and WR are commonly reported long-term complications after bariatric procedures, with higher incidences among restrictive procedures such as LSG [16]. High revision rates after LSG have been reported in the literature [14]. In this regard, revision rates up to 10% and 22% in studies with follow-up durations of ≥ 3 years and ≥ 10 years, respectively, have been observed [3].

RYGB is the most commonly performed revision procedure after failed LSG [3, 5]. This observation may be explained by the favorable outcomes of RYGB after LSG, including satisfactory weight loss effect and less serious nutritional deficiencies [14, 15]. Studies have reported good additional weight loss effect after revisional RYGB for failed LSG, additional resolution of associated medical problems, and a 75% improvement in reflux symptoms [14, 15]. Furthermore, some studies reported good results regarding GERD resolution, with no noteworthy additional weight loss after revisional RYGB for failed LSG [17]. In the literature, OAGB is still less commonly used than RYGB after LSG [3, 5]. However, the popularity of primary OAGB has grown over the last decade, reaching 7.6% of all bariatric procedures performed worldwide in 2018 [18]. Compared to RYGB, the exponents of OAGB showed superior weight loss effect and lower complication rates, in addition its simpler technique, shorter procedure time, and shorter learning curve [8, 19].

The relation between the length of the biliopancreatic limb in RYGB and weight loss has been a matter of debate in the literature. At the same time, data from some studies and systematic reviews refer to a higher weight loss with the longer biliopancreatic limbs. Data from large volume and prospective studies reported no significant differences between longer and shorter biliopancreatic limbs in the weight loss [20–22].

Nergaard et al. compared a long biliopancreatic limb (200 cm) with a short alimentary limb (60 cm) to a short biliopancreatic limb (60 cm) with a long alimentary limb (150 cm) in RYGB and reported significantly higher total weight loss and %EBMIL with the longer biliopancreatic limb that continued for 7 years after surgery. However, the longer biliopancreatic limb was associated with more malabsorption and significantly more need for supplementation [23]. Some other authors compared a short biliopancreatic limb (70 cm) to a longer biliopancreatic limb (120 cm) with a fixed alimentary limb length (150 cm) and reported comparable %EBMIL and remission of associated medical problems between the two groups during 5 years of follow-up. Moreover, the longer biliopancreatic limb was associated with more need for supplementation of vitamins B12, A, and folic acid [22].

The same debate exists in OAGB; some authors use a fixed biliopancreatic limb length of 200 cm, [24], whereby other authors recommend a biliopancreatic limb length of 150 cm to avoid severe nutritional deficiencies [25]. Moreover, some authors proposed tailoring of the biliopancreatic limb in RYGB according to the BMI of the patients using a length of 60 cm if BMI < 45, 80 cm if BMI is between 45 and 50, and 100 cm if BMI > 50. Similarly, in OAGB, they proposed using a biliopancreatic limb length of 120 cm for BMI < 45, 200 cm for BMI between 45 and 50, and 220 cm for BMI > 50 [26]. In this study, we recorded excellent weight loss and nutritional laboratory values between the OAGB group with a 200-cm biliopancreatic limb length and the RYGB group with a biliopancreatic limb length of 100 cm combined with a 100-cm alimentary limb through 2 years of follow-up.

### BMI and Weight Loss

In this study, both OAGB and RYGB groups showed significant BMI reductions after revision surgery. BMI decreased from 44.9 ± 6.6 kg/m^2^ and 45.1 ± 8.3 kg/m^2^ before revision surgery to 27.8 ± 2.2 kg/m^2^ and 27.4 ± 3.1 kg/m^2^ 2 years after revision surgery in the RYGB and OAGB groups, respectively (*p* < 0.0001). In addition, both groups achieved BMI values that were significantly lower than their post-LSG nadir BMI (*p* < 0.001).

The weight loss effects of both groups were almost equal. Data available from some retrospective comparisons of RYGB to OAGB after LSG showed significantly higher weight loss effects in the OAGB group than in the RYGB group [8, 27]. However, other studies have reported similar weight loss effects with both procedures [28]. The more favorable weight loss effect in OAGB might be correlated with more malabsorption due to longer biliopancreatic limbs. Increasing the length of the biliopancreatic limb was associated with improved weight loss and a higher risk of malnutrition [24].

Since OAGB should not be recommended for patients with higher grades of GERD [29], these patients were excluded from this study to allow proper randomization between RYGB and OAGB groups. Moreover, recent comparisons of OAGB to RYGB after LSG showed significantly lower rates of resolution of GERD after OAGB than after RYGB [27, 28].

### Operation Time

The mean operative time in the RYGB group (104.9 ± 13.7 min) was significantly higher than that of the OAGB group (85.6 ± 18.6 min) (*p* < 0.001). OAGB is simpler than RYGB, with only one anastomosis. The simplicity of the technique is also an important issue, as some surgeons would prefer re-sleeve, a less technically demanding technique, over RYGB for failed LSG despite the questionable effectiveness of long-term weight loss maintenance [30].

### Complications

The overall complication rates were comparable between the OAGB and RYGB groups in this study, which was consistent with previously published data [29, 31]. This study showed no leaks in the OAGB group and one (1.3%) case of leak in the RYGB group. The leakage was successfully managed with a self-expandable metallic stent (SEMS) for 2 weeks. SEMS can be used safely for early leaks after RYGB, with high success rates [32].

Bleeding requiring intervention occurred in one patient (1.3%) in each group. Internal hernia occurred in one (1.3%) patient in the RYGB group, despite routine closure of the mesenteric defects, while port-site hernias occurred in one (1.3%) patient in each group, all of whom needed reintervention. Bleeding, intestinal obstruction, and leakage are common causes of reintervention after RYGB [33]. While OAGB has a lower incidence of internal hernia than RYGB, intestinal obstruction can still occur due to port-site hernia and less often due to internal hernia [34].

### GERD

In both groups of this study, all patients with symptomatic reflux before revision surgery had their symptoms relieved after this procedure and stopped their medications. De novo grade A GERD [12] was diagnosed in one (1.3%) patient with RYGB (acid reflux), while bile reflux was diagnosed in two (2.5%) patients in the OAGB group. Patients in both groups were managed with medical treatment, which included proton pump inhibitors and prokinetic-s as mosapride, and for bile reflux, sucralfate, and ursodeoxycholic acid were added to the treatment. RYGB has always been considered the optimal treatment option for patients with GERD, with reportedly high rates of GERD resolution [7, 17, 27, 28].

### Bile Reflux

However, OAGB is not the preferred option for patients with GERD due to concerns of bile reflux. Long-term data on GERD after primary and revisional OAGB is currently not available [7]. Despite the inherent concerns about bile reflux after OAGB, evidence from large OAGB series has shown that the incidence is lower than the first feared (0.4–1.8%) [35, 36]. Still, bile reflux remains the predominant cause of re-operation following OAGB [37]. The incidence in our study was 2.5%, without need for reoperation. Several surgical solutions are available when medical treatment failed including conversion to RYGB, Braun anastomosis and dividing the afferent limb, and re-anastomosing it to the efferent limb downstream [38]. This study showed a low incidence of bile and acid reflux in both OAGB and RYGB groups, with no significant differences. However, it is worth noting that the study power was insufficient to detect any reflux for the possibility to generate a scientific model in deciding which operation would be the best for reflux prevention in the future.

### Marginal Ulcers

In this study, marginal ulcers (MU) were diagnosed by endoscopy in two (2.5%) patients in the RYGB group and no patients in the OAGB group, with no significant differences. Medical treatment was effective in both patients with RYGB. Some authors reported a higher incidence of MU in revisional OAGB (17.6%) than in revisional RYGB (9.5%) [8]. However, the reported incidence of MU was approximately 4.6% in RYGB, ranging from 1 to 9%, whereas that of OAGB was approximately 2.4%, ranging from 1.6 to 17.6% [7, 39]. Our observed rates of MU diagnosis were lower than these reported rates. However, endoscopy was performed only for patients with symptoms and was not routinely performed during the follow-up period of this study. Therefore, we might have missed some cases with GERD or MU.

### Associated Medical Problems

Both groups in this study experienced comparable resolution and/or improvement of associated medical problems. Additionally, we observed a pattern of improvement or resolution of dyslipidemia, hypertension, and type 2 diabetes. Consistent with our findings, previous studies showed that both revisional RYGB and OAGB had high rates of resolution and/or improvement of associated medical problems, which were comparable to each other and to those of primary procedures [3, 7, 8, 14, 15, 17, 19, 28–31].

### Nutritional Values

Laboratory nutritional values were comparable in both groups in this study. Patients in both groups were prescribed the same multi-vitamins and mineral supplements. Some authors reported a higher incidence of nutritional deficiencies, such as iron deficiency anemia, in OAGB than in RYGB [19]. The incidence of nutritional deficiencies may be correlated with the malabsorptive effect of surgery, which may depend on the length of the biliopancreatic limb or the combined length of the bypassed small bowel [24].

### Optimal Procedure

Regarding the optimal procedure for patients with WR or WLF with GERD grade A, both OAGB and RYGB showed good outcomes for weight loss and incidence of de novo GERD or MU. However, a longer follow-up (> 2 years) should provide more insights on all variables, possible weight regain after revision surgery, and malnutrition in OAGB. In general, both types of revision surgery were safe and effective, providing the surgeons with good opportunities to determine the optimal treatment together with the patient. Since patients with GERD grade B or higher were not included in our study, RYGB remained the preferred operation, as mentioned in the literature [39].

### Strengths and Limitations

To our best knowledge, this is the first randomized controlled trial to compare RYGB and OAGB as revision procedures for WR after LSG. However, the study had some limitations. First, while the study was powered to detect an effect size corresponding to a %EBMIL difference of approximately 10% between RYGB and OAGB, a larger sample is needed to detect potentially more subtle differences between and within these two groups. Second, the study had a short-term follow-up of 2 years, and a longer follow-up duration is needed. Third, patients with higher grades of GERD were excluded from the study; therefore, the impact of OAGB on higher grades of GERD remains still unknown. Fourth, routine endoscopy was not attempted after the revision procedures, resulting in possible missing data regarding GERD and MU in our study. Fifth, data about smoking cessation was not recorded in the follow-up; this may affect MU rates. Sixth, we did not have the complete records of all patients for the original LSG as around half of them had their original LSG in other centers, so we did not include data about improvement/resolution of associated medical conditions after the primary LSG procedure in the study.

## Conclusion

Both revisional RYGB and OAGB had comparable significant weight loss effects when performed for WR after LSG, with comparable resolution or improvement of associated medical problems. Given the advantage of being a simpler and easier procedure, OAGB may have a greater chance of being the optimal revision procedure if longer follow-up data are available. After a 2-year follow-up, both procedures were safe, with no significant differences in the occurrence of complications and nutritional deficits.

## Appendix 1

Table 4

**Table 4:** CONSORT 2010 checklist of information to include when reporting a randomised trial*****

Section/topic	Item no	Checklist item	Reported on page no.
Title and abstract			
	1a	Identification as a randomized trial in the title	Page 1
	1b	Structured summary of trial design, methods, results, and conclusions (for specific guidance see CONSORT for abstracts)	Page 1
Introduction			
Background and objectives	2a	Scientific background and explanation of rationale	Page 3
	2b	Specific objectives or hypotheses	Page 3
Methods			
Trial design	3a	Description of trial design (such as parallel, factorial) including allocation ratio	Page 4
	3b	Important changes to methods after trial commencement (such as eligibility criteria), with reasons	Page 4
Participants	4a	Eligibility criteria for participants	Page 4–5
	4b	Settings and locations where the data were collected	Page 4
Interventions	5	The interventions for each group with sufficient details to allow replication, including how and when they were actually administered	Page 7
Outcomes	6a	Completely defined pre-specified primary and secondary outcome measures, including how and when they were assessed	Page 7–8
	6b	Any changes to trial outcomes after the trial commenced, with reasons	Page 7–8
Sample size	7a	How sample size was determined	Page 9
	7b	When applicable, explanation of any interim analyses and stopping guidelines	n.a
Randomization			
Sequence generation	8a	Method used to generate the random allocation sequence	Page 9
	8b	Type of randomication; details of any restriction (such as blocking and block size)	Page 9
Allocation concealment mechanism	9	Mechanism used to implement the random allocation sequence (such as sequentially numbered containers), describing any steps taken to conceal the sequence until interventions were assigned	Page 9–10
Implementation	10	Who generated the random allocation sequence, who enrolled participants, and who assigned participants to interventions	Page 9–10
Blinding	11a	If done, who was blinded after assignment to interventions (for example, participants, care providers, those assessing outcomes) and how	Page 9
	11b	If relevant, description of the similarity of interventions	Page 8–9
Statistical methods	12a	Statistical methods used to compare groups for primary and secondary outcomes	Page 8–9
	12b	Methods for additional analyses, such as subgroup analyses and adjusted analyses	Page 8–9
Results			
Participant flow (a diagram is strongly recommended)	13a	For each group, the numbers of participants who were randomly assigned, received intended treatment, and were analysed for the primary outcome	Page 11
	13b	For each group, losses and exclusions after randomisation, together with reasons	Page 11
Recruitment	14a	Dates defining the periods of recruitment and follow-up	Page 11
	14b	Why the trial ended or was stopped	n.a
Baseline data	15	A table showing baseline demographic and clinical characteristics for each group	Page 11
Numbers analysed	16	For each group, number of participants (denominator) included in each analysis and whether the analysis was by original assigned groups	Page 11–13
Outcomes and estimation	17a	For each primary and secondary outcome, results for each group, and the estimated effect size and its precision (such as 95% confidence interval)	Page 11–13
	17b	For binary outcomes, presentation of both absolute and relative effect sizes is recommended	Page 11–13
Ancillary analyses	18	Results of any other analyses performed, including subgroup analyses and adjusted analyses, distinguishing pre-specified from exploratory	Page 11–13
Harms	19	All important harms or unintended effects in each group (for specific guidance see CONSORT for harms)	Page 11–13
Discussion			
Limitations	20	Trial limitations, addressing sources of potential bias, imprecision, and, if relevant, multiplicity of analyses	Page 14–21
Generalizability	21	Generalizability (external validity, applicability) of the trial findings	Page 14–21
Interpretation	22	Interpretation consistent with results, balancing benefits and harms, and considering other relevant evidence	Page 14–21
Other information			
Registration	23	Registration number and name of trial registry	Page 5
Protocol	24	Where the full trial protocol can be accessed, if available	corresponding-author
Funding	25	Sources of funding and other support (such as supply of drugs), role of funders	Page 22

*We strongly recommend reading this statement in conjunction with the CONSORT 2010 Explanation and Elaboration for important clarifications on all the items. If relevant, we also recommend reading CONSORT extensions for cluster randomised trials, non-inferiority and equivalence trials, non-pharmacological treatments, herbal interventions, and pragmatic trials. Additional extensions are forthcoming: for those and for up-to-date references relevant to this checklist, see www.consort-statement.org

## Appendix 2 Surgical techniques

### The RYGB Operation

Standard 5 ports were used including three 12-mm ports (for the camera, right and left working ports) and two 5-mm ports (for liver retraction and for the assisstant). Pneumo-peritoneum was created after using optical trocars for entery paying attention to the presence of adhesions from previous surgery. Starting with dissection of adhesions around the gastric sleeve using the energy device EnSeal® (Ethicon Endo-Surgery, Cincinnati, OH, USA). The gastric pouch was created starting at 5–6 cm below the esophago-gastric junction using Echelon Flex Endopath 60-mm linear stapler (Ethicon Endo-Surgery, Cincinnati, OH, USA) over a 40 fr bougie, using gold and blue reloads. The same stapler was used for construction of the gastro-jejunostomy and the jejuno-jejunostomy using blue and white reloads, respectively, with equal 100-cm bilio-pancreatic and alimentary limbs. The stapling defects were closed using barbed sutures, 3/0 V-Loc 180 sutures (Covidien, Mansfield, MA, USA) in two layers. The staple line in the gastric pouch and the remnant stomach were reinforced with seromuscular continuous sutures using the same barbed sutures. All mesenteric defects were closed using 3/0 V-Loc non-absorbable sutures (Covidien, Mansfield, MA, USA).

### The OAGB Operation

Same 5 ports were used. The creation of the long gastric pouch started at the crow’s foot on the lesser curvature, using the same linear stapler, over a 40 fr bougie, using gold and blue reloads. Blue reloads were used for construction of the gastro-jejunostomy, at 200 cm from the ligament of Treitz. Closure of the stapling defects and staple line reinforcement were done in the same way as in RYGB. Closure of mesenteric defects was not needed in OAGB.

Crural repair for hiatal hernia using 2/0 V-Loc non-absorbable sutures (Covidien, Mansfield, MA, USA) was attempted in all cases with pre-operatively diagnosed hiatal hernia in both groups. Concomitant cholecystectomy was performed in in all cases with pre-operatively diagnosed calcular cholecystitis in both groups. Intra-operative methylene blue leak test was routinely performed. A tube drain was routinely placed in the left sub-phrenic space.
